# Factors associated with depression and cognitive impairment after cerebral hemorrhage surgery

**DOI:** 10.1038/s41598-024-53437-x

**Published:** 2025-11-17

**Authors:** Bin Wen, Ping Zhang, Xiang Niu, Hangtian Cheng, Zhong Li, Mingjie Zhang, Ziyu Zhao

**Affiliations:** 1 Department of Neurosurgery, Tianshui First People’s Hospital, No. 105 Construction Road, Tianshui, 741000 Gansu People’s Republic of China; 2 Department of Neurosurgery, Gansu University of Chinese Medicine Affiliated Tianshui Hospital, No. 105 Construction Road, Tianshui, 741000 Gansu People’s Republic of China; 3 Department of TCM, Tianshui First People’s Hospital, No. 105 Construction Road, Tianshui, 741000 Gansu People’s Republic of China

**Keywords:** Neuroscience, Psychology, Diseases, Neurology

## Abstract

This study aims to investigate various factors, such as hemorrhage locations, cognitive and emotional outcomes, to provide valuable information for clinical interventions and the management of mental disorder patients following surgical procedures. A total of 94 patients who underwent surgery were included, and their demographic information, encompassing surgical methods, pre- and post-surgical haemorrhagic data, Then mobility of limbs and psychological assessments were collected. At 2 weeks post-surgery, the HAMD score for the right Basal Ganglia Haemorrhage (BGH) group was significantly higher than that of the right Basal Ganglia Breaking into ventricular haemorrhage (BGBVH), ventricular infarction and haemorrhage (VIH), or cerebellar haemorrhage (CLH) groups (all *P* < 0.05). At 3 months, there was a significant difference in HAMD score between the high-risk right BGH and the low-risk VIH groups (*P* = 0.023). There was a correlation between functional independence measure (FMA), activities of daily living (ADL) and HAMD scores, as well as a linear relationship between Glasgow Coma Scale (GCS)/ADL scores and Mini-Mental State Examination (MMSE) scores, and a linear relationship between haemorrhage location, ADL score, and Montreal Cognitive Assessment (MoCA) scores. The primary factor contributing to depression in patients with intracerebral haemorrhage (ICH) is the decrease in ADL and FMA scores after surgery. Additionally, ADL, GCS, surgical methods and bleeding sites can affect the cognitive function of patients.

## Introduction

Hypertensive people with cerebral hemorrhage account for up to 51.5%^1^ or 20%^2^ of strokes in China or Asia^3^. Approximately 50% of cerebral hemorrhage patients with hypertension die within 48 h of onset, and the mortality rate exceeds 50% 1 year following medical intervention^1^. However, the mortality has been significantly reduced in post-operative cerebral hemorrhage patients, likely due to major improvements in intracranial hemorrhagic (ICH) neurosurgical intervention^1^. Despite this progress, 80% of ICH survivors experience physical disability^1–3^, and mental state and cognitive function are significantly impacted among ICH patients, particularly in non-operative microvascular hemorrhagic patients^4^.

Cerebral microbleeds (CMBs) have received extensive attention due to the relatively long survival time and good follow-up of CMB patients, with up to 23% of survivors of cerebral hemorrhage presenting with post-stroke depression within 1 year^5,6^. Female, hispanic ethnicity, right-side ICH with intraventricular hemorrhage (IVH), cognitive dysfunction, and non-family residence on day 30 were independently related to post-stroke depression (PSD) on day 180 post-onset, and 40% of these ICH survivors have significant depression^6^.

Hypertensive cerebral hemorrhage can result in emotional and cognitive dysfunction, which is a chronic and progressive condition characterized by decline or disappearance of cognition, memory, speech, emotion, behavior, and personality. Depressed patients may present with symptoms such as fatigue, pain, anxiety, pessimism, despair, sleep disorders, and reluctance to eat or receive treatment^7–9^. The factors influencing mental status post-surgery for these patients and the impact of different surgical approaches, bleeding sites, rehabilitation, and complications on mental state remain to be explored. Therefore, this study aims to investigate these outcomes to provide useful information for clinical interventions and the management of mental disorder patients following surgical interventions.

## Data and methods

### Research object

Upon admission, medical history and physical examination data were collected, including assessment of baseline consciousness level, using the Glasgow Coma Scale (GCS), determination of symptom onset time, and calculation of the "visiting time" from symptom onset to entering the operating room.

The selection criteria were as follows: (1) definite history of hypertension, meeting the diagnostic criteria of the fourth national working conference on cerebral hemorrhage; (2) first cerebral hemorrhage confirmed by CT scan with surgical indication, with supratentorial or infratentorial ICH volume > 30 ml or 10 ml, respectively, and ventricular hemorrhage with casting mold reaching surgical indication.

Exclusion criteria were: (1) CT showing potential structural vascular abnormalities such as arteriovenous malformation or aneurysm (with subarachnoid hemorrhage); (2) severe systemic diseases, end-stage medical diseases, coagulopathy, traumatic ICH, pregnancy, severe aphasia, mental disability, or inability to provide informed consent or interfere with outcome evaluation; (3) late stage of cerebral hernia, near-death state, clear surgical taboo, or refusal of surgery; (4) self-discharge.

### Clinical data collection

There are many factors that influence the emotional and cognitive dysfunction, and we have maximum possible inclusion of all clinical factors. Demographic and clinical information, encompassing surgical methods, pre- and post-surgical haemorrhagic data, Then mobility of limbs and psychological assessments were collected. Pre-surgical haemorrhagic data included age, sex, Glasgow Coma Scale (GCS), time from symptom onset to surgery, presence of Temporal Sulcus Hernia (TSH), and the bleeding location within the cerebral lobe (BGH, VIH, cerebellum, CLH, BGBVH). Surgical methods were categorized as I (Craniotomy hematoma evacuation and decompressive craniectomy), II (Cranial flap reset with a small bone window), or III (Hematoma puncture and external drainage). Post-surgical haemorrhagic data included blood pressure, post-operative rehabilitation, and post-surgery complications such as aspiration pneumonia and stress ulcer. Psychological and mobility of limbs assessments (MMSE, HAMD, MoCA, ADL, and FMA) were conducted at 2 weeks, 1 month, and 3 months.

### Surgical methods

**Figure 1:**
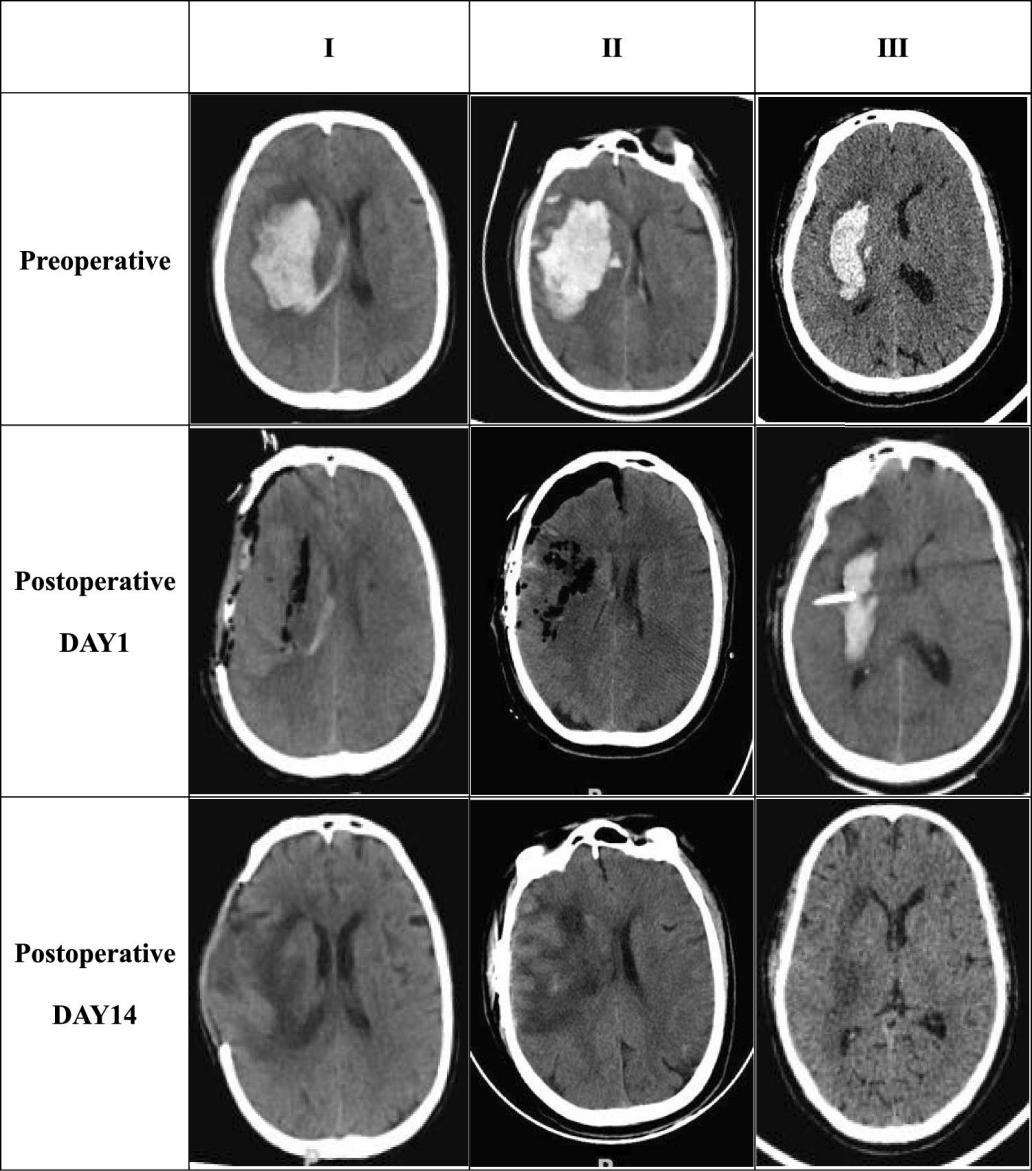
CT manifestations of different surgical approaches in the right BGH.

1. Craniotomy hematoma evacuation and decompressive craniectomy: choose frontoparietal–temporoparietal "?" Curved incision. The cranial window is approximately (7–10) cm × (9–12) cm in size. Regarding hemorrhage on the surface of the cerebral lobe, such as the occipital lobe and frontal lobe, a horseshoe-shaped incision was used for craniotomy. The cranial window is approximately 8 cm × 10 cm in size. After removing the hematoma, decompressive craniectomy was performed^10,11^.
2. Cranial flap reduction with a small bone window: a bone window with a diameter of approximately 4 cm is cut, and the hematoma is removed before bone flap reposition.
3. Hematoma puncture and external drainage: according to the hematoma shown by cranium CT, calculate the superficial position of the hematoma from the scalp, cut the scalp, drill the skull, open the dura mater, puncture the hematoma cavity with a lateral ventricle drainage tube, and place the drainage tube. Lateral intraventricular drainage: after anesthesia, 1.5 cm in front of the coronal suture of the forehead and 2.5 cm beside the midline were selected as puncture points. Conventional puncture was performed, and an external drainage tube was connected. 20,000 units of Urokinase were injected into the hematoma cavity on the second day after the operation, and the drainage tube was opened after 4 h for fibrinolysis^11,12^ (Fig. 1)

### Mental state evaluation

#### Mini-Mental State Examination (MMSE)

MMSE is a widely used tool for screening cognitive impairment due to its simplicity and ability to be completed within 10 min^13^ to evaluate overall cognitive function, which includes orientation (10), immediate recall (3), attention and calculation (5), delayed recall (3), and language (9). The highest total score is 30. Based on MMSE guidelines, scores can be divided into two categories: cognitive normal (MMSE > 24) and cognitive impaired (MMSE < 24)^14,15^.

#### Montreal Cognitive Assessment Scale (MOCA)

The MOCA to measure cognitive state, including comprehension, reading, writing, orientation, and drawing abilities. However, there are doubts about its effectiveness in accurately measuring these subgroups^16^. In China, MoCA has been found to be more effective than the MMSE in predicting vascular cognitive dysfunction (0.667 vs. 0.626)^13^. In cases where patients are unable to remember and accurately answer questions, only three-step command scores are recorded^17^. The evaluation assesses the following six parameters: memory, visual space ability, executive function, attention and concentration, language, and orientation. The maximum score is 30^10^, and a score of ≤ 25 indicates cognitive impairment^18^.

#### Hamilton Depression Scale (HAMD)

HAMD is a reliable tool for evaluating depression^19^. It consists of 21 questions, but only the first 17 questions are scored. Eight items are rated from 0 (non-existent) to 4 (severe), and nine items are rated from 0 to 2. Patients with a score of 8 or above are classified as depressed^20^.

### Statistical treatment

SPSS 23.0 software was used for data analysis. For normally distributed measurement data, the mean and standard deviation (${\overline{\text{x}}}$ ± S) were calculated. Pairwise comparisons between groups were conducted using the LSD method if repeated measurement data followed a normal distribution and met the spherical hypothesis test. If the test did not meet the spherical hypothesis, the adjusted Greenhouse test was used.

### Ethical approval

The questionnaire and methodology for this study were approved by the Human Research Ethics Committee of the Tianshui First People’s Hospital (Ethics approval number: Tian Ethics 20180106). The authors assert that all procedures contributing to this work comply with the ethical standards of the relevant national and institutional committees on human experimentation and with the Helsinki Declaration of 1975, as revised in 2008. Informed consent was obtained from all individual participants included in the study and consent to publish.

## Results

### Basic information

A total of 221 patients with cerebral hemorrhage were initially included. However, due to language barriers resulting from hemorrhages in the left basal area, only 94 patients were included in the final study, comprising of 49 males (52.1%) and 45 females (47.9%), with an average age of 54.91 ± 9.6 years. Of the included patients, 31 (33%) underwent surgery using method I, 23 (24.5%) underwent surgery using method II, and 40 (42.6%) underwent surgery using method III. Additionally, 53 patients (56.4%) experienced pulmonary infections, and 62 patients (72.3%) received rehabilitation within one week of hospital admission (Table 1).

**Table 1:** Comparison of various influencing factors between cognitive impairment and cognitive normal HAMD scale score at 3 months.

	Impairment 42 (> 8)	Normal 52 (≤ 7)	*t/Z/χ*^*2*^	*P* value
Gender (A)				
Female (n%)	28 (53.8)	17 (40.5)	1.664	0.219
Age (B)				
${\overline{\text{x}}}$ ± S	54.38 ± 10.31	55.35 ± 9.07	0.483	0.631
Onset surgery time (C)				
Median (*P*_25,_ *P*_75_)	6 (5, 9)	5 (4,8)	1.99	0.158
GCS (D)				
${\overline{\text{x}}}$ ± S	8.88 ± 2.91	9.15 ± 2.16	1.372	0.174
Brain herniation (E)				
No (n%)	39 (92.9)	47 (90.4)	0.03	0.956
Surgical method (F)				
I (n%)	16 (38.1)	15 (28.8)		
II	11 (26.2)	12 (23.1)		
III	15 (35.7)	25 (48.1)	1.529	0.502
Postoperative BP (G)				
SBP median (*P*_25,_*P*_75_)	158 (137, 169)	152 (142, 169.25)	− 0.240	0.811
DBP	91 (82, 99.75)	90 (80.75, 98.25)	− 0.563	0.576
Rehabilitation (H)				
Yes (n%)	31 (73.8)	37 (71.2)	0.082	0.820
Complication (L)				
Upper GI bleeding (n%)	0	2 (3.8)		
Pulmonary infection	22 (52.4)	31 (59.6)	2.145	0.42
Haemorrhage location (J)				
Right BGH (n%)	23 (54.8)	18 (34.6)		
Right BGBVH	7 (16.7)	13 (25)		
VIH	4 (9.5)	14 (26.9)		
Cerebellum	5 (11.9)	5 (9.6)		
CLH	3 (7.1)	2 (3.8)	7.231	0.118
ADL (K)				
Median (*P*_25,_ *P*_75_)	50 (30, 80)	60 (41.25, 100)	5.031	0.025
FMA (L)				
Median (*P*_25,_*P*_75_)	20 (10, 35.5)	35 (11.5, 95)	4.383	0.036

### Correlation analysis between HAMD scale score and various factors.

The study conducted a HAMD (Hamilton Rating Scale for Depression) assessment on patients 3 months post-surgery to determine their depression status. Fifty two out of the 94 patients were categorized as normal; while 42 were identified as depressed.

To investigate the differences between the two groups mentioned above, several factors were compared. These included sex, age, visiting time, GCS (Glasgow Coma Scale) score, brain herniation status (temporal lobe gyrus herniation), bleeding location, operation method, postoperative blood pressure, post-operative rehabilitation status, post-surgery complications, and ADL (Activity of Daily Living) and FMA (Fugl–Meyer Assessment) scores. The median ADL score was found to be 50 (P25 30, P75 80) in the depression group and 60 (P25 41.25, P75 100) in the normal group, 3 months after surgery. A significant difference was observed between the two groups (Z = 5.031, P = 0.025). Similarly, the median FMA score was 20 (P25 10, P75 35.5) in the HAMD cognitive dysfunction group and 35 (P25 11.25, P75 95) in the normal group, and a significant difference was observed (Z = 4.383, P = 0.036) (Table 1).

Furthermore, the study conducted a linear regression analysis of ADL and FAM in the HAMD depression group. The results indicated that both FMA score (B = 0.043, t = 2.396, P = 0.021) and ADL score (B = − 0.049, t = − 2.308, P = 0.026) were linearly related to the HAMD score. The regression equation obtained was y = 11.997 + 0.043L − 0.049K (Table 2). This indicates that both FMA and ADL scores can be used as reliable indicators of depression severity in patients after hypertensive cerebral hemorrhage.

**Table 2:** Correlation analysis between HAMD scale scores and various factors.

Model	Unstandardized regression coefficient		*t*	*P*	95.0% confidence interval for B	
	B	Std. error			Lower bound	Upper bound
(Constant)	11.997	0.869	13.799	0.000	10.238	13.755
FMA3	0.043	0.018	2.396	0.021	0.007	0.079
ADL3	− 0.049	0.021	− 2.308	0.026	− 0.092	− 0.006

### Correlation analysis between MMSE scale score and various factors

The study assessed patients using the MMSE score 3 months post-surgery and categorized them into two groups based on their scores. A total of 40 patients with scores > 25 were classified as normal, while 54 patients with scores ≤ 24 were classified as depressed. To compare the cognitive function between the two groups, the study analyzed GCS (Glasgow Coma Scale), ADL (Activity of Daily Living), and FMA (Fugl–Meyer Assessment) scores. The GCS score was found to be 8 (P25 6, P75 11) in the MMSE cognitive dysfunction group and 10.5 (P25 8, P75 12.5) in the normal group. A significant difference was observed between the two groups (Z = − 2.413, P = 0.016).

The ADL score was also compared between the two groups and was found to be 40 (P25 35, P75 60) in the MMSE cognitive dysfunction group and 97 (P25 60, P75 100) in the normal group. A significant difference was observed between the two groups (Z = − 6.596, P = 0.001). Similarly, the FMA score was found to be 15 (P25 9, P75 20) in the MMSE cognitive impairment group and 90 (P25 35.5, P75 100) in the normal group, with a significant difference (Z = − 5.371, P = 0.001) (Table 3). Additionally, a linear regression equation was used to analyze the GCS, ADL, and FAM scores in the MMSE cognitive impairment score group. The results showed that the GCS score (B = 0.543, t = 2.061, P = 0.044) and ADL score (B = − 0.195, t = 3.812, P = 0.001) were linearly related to the MMSE score in the depression group. The regression equation obtained was y = − 2.413 + 0.543D − 0.195K (Table 4). These results suggest that both GCS and ADL scores can serve as reliable indicators of cognitive impairment severity in patients with depression after surgery.

**Table 3:** Comparison of various influencing factors between cognitive impairment and cognitive normal MMSE scale score at 3 months.

	Impairment 54 (≤ 24)	Normal 40 (> 24)	*t/Z/χ*^*2*^	*P* value
Gender (A)				
Female (n%)	24 (44.4)	21 (52.5)	0.598	0.532
Age (B)				
${\overline{\text{x}}}$ ± S	55.48 ± 10.81	54.15 ± 7.76	0.695	0.489
Onset surgery time (C)				
Median (*P*_25_, *P*_75_)	6 (4, 7)	6 (4, 12)	− 0.528	0.597
GCS (D)				
Median (*P*_25_, *P*_75_)	8 (6, 11)	10.5 (8, 12.5)	− 2.413	0.016
Brain herniation (E)				
No (n%)	47 (87)	39 (97.5)	2.027	0.154
Surgical method (F)				
I (n%)	23 (42.6)	8 (20)		
II	10 (18.5)	13 (32.5)		
III	21 (38.9)	19 (47.5)	5.793	0.055
Postoperative BP (G)				
SBP ${\overline{\text{x}}}$ ± S	152.28 ± 19.88	156.28 ± 21.80	− 0.925	0.357
DBP ${\overline{\text{x}}}$ ± S	89.17 ± 16.68	93.27 ± 13.36	− 1.282	0.203
Rehabilitation (H)				
Yes (n%)	40 (74.1)	28 (70)	0.191	0.816
Complication (L)				
Upper GI bleeding (n%)	0	2 (5)		
Pulmonary infection	29 (56.7)	24 (60)	3.146	0.159
Hemorrhage location (J)				
Right BGH (n%)	28 (51.9)	13 (32.5)		
Right BGBVH	13 (24.1)	7 (17.5)		
VIH	8 (14.8)	10 (25)		
Cerebellum	3 (5.6)	7 (17.5)		
CLH	2 (3.7)	3 (7.5)	7.302	0.113
ADL (K)				
Median (*P*_25_, *P*_75_)	40 (35,60)	97 (60, 100)	− 6.569	0.001
FMA (L)				
Median (*P*_25_, *P*_75_)	15 (9, 20)	90 (35.5, 100)	− 5.371	0.001

**Table 4:** Correlation analysis between MMSE scale scores and various factors.

Model	Unstandardized regression coefficient		*t*	*P*	95.0% confidence interval for B	
	B	Std. error			Lower bound	Upper bound
(Constant)	− 2.413	2.735	− 0.882	0.382	− 7.905	3.080
GCS	0.543	0.263	2.061	0.044	0.014	1.072
FMA3	0.006	0.051	0.108	0.914	− 0.097	0.108
ADL3	0.195	0.051	3.812	0.000	0.092	0.297

### Correlation analysis between MoCA scale scores and various factors

According to the MoCA score at 3 months post-surgery, 25 patients with a score ≥ 26 points were classified as normal, while 69 patients with a score ≤ 25 points were classified as cognitively impaired. There was a significant difference in bleeding location distribution between the normal and cognitive impairment groups after 3 months of hypertensive cerebral hemorrhage (χ^2^ = 9.091, P = 0.045). The ADL score was 20 (P25 10, P75 35) or 95 (P25 37, P75 100) in the cognitive impairment or normal group, respectively (Z = − 6.128, P = 0.001). The FMA score was 15 (P25 9, P75 20) or 90 (P25 35.5, P75 100) in the cognitive impairment or normal group, respectively (Z = − 5.175, P = 0.001) (Table 5). A multiple linear regression equation was applied to bleeding location, ADL score, and FAM score in the MoCA cognitive impairment score group. The results showed that bleeding location (B = − 1.397, t = − 3.241, P = 0.002), ADL score (B = 0.225, t = 4.876, P = 0.001), and cognitive impairment were significantly related to each other. The regression equation is y = 5.389 − 1.397J + 0.225K (Table 6).

**Table 5:** Comparison of various influencing factors between cognitive impairment and cognitive normal MoCA scale score at 3 months.

	Impairment 69 (≤ 25)	Normal 25 (> 26)	*t/Z/χ*^*2*^	*P* value
Gender (A)				
Female (n%)	30 (43.5)	15 (60)	2.007	0.170
Age (B)				
${\overline{\text{x}}}$ ± S	54.97 ± 10.44	54.76 ± 6.98	0.112	0.911
Onset surgery time (C)				
Median (*P*_25_, *P*_75_)	6 (4, 8)	6 (4, 13)	− 0.414	0.682
GCS (D)				
${\overline{\text{x}}}$ ± S	9.07 ± 3.13	10.16 ± 2.79	− 1.529	0.130
Brain herniation (E)				
No (n%)	61 (88.4)	25 (100)	1.854	0.173
Surgical method (F)				
I (n%)	26 (37.7)	5 (20)		
II	17 (24.6)	6 (24)		
III	26 (37.7)	14 (56)	3.19	0.200
Postoperative BP (G)				
SBP median (*P*_25_, *P*_75_)	152.81 ± 20.76	157.20 ± 20.61	− 0.907	0.367
DBP	90.38 ± 16.55	92.40 ± 11.89	− 0.56	0.577
Rehabilitation (H)				
Yes (n%)	51 (73.9)	17 (68)	0.321	0.607
Complication (L)				
Upper GI bleeding (n%)	1 (1.4)	1 (4)		
Pulmonary infection	37 (53.6)	16 (64)	2.028	0.287
Haemorrhage location (J)				
Right BGH (n%)	35 (50.7)	6 (24)		
Right BGBVH	15 (21.7)	5 (20)		
VIH	12 (17.4)	6 (24)		
Cerebellum	5(7.2)	5 (20)		
CLH	2(2.9)	3 (12)	9.091	0.045
ADL (K)				
Median (*P*_25_, *P*_75_)	20 (10, 35)	95 (37, 100)	− 6.128	0.001
FMA (L)				
Median (*P*_25_, *P*_75_)	15 (9, 20)	90 (35.5, 100)	− 5.175	0.001

**Table 6:** Correlation analysis between MoCA scale scores and various factors.

Model	Unstandardized regression coefficient		*t*	*P*	95.0% confidence interval for B	
	B	Std. error			Lower bound	Upper bound
(Constant)	5.389	2.055	2.622	0.011	1.284	9.493
Haemorrhage location	− 1.397	0.431	− 3.241	0.002	− 2.257	− 0.536
FAM3	0.039	0.036	1.075	0.286	− 0.033	0.111
ADL3	0.225	0.046	4.876	0.000	0.133	0.317

### Effect of surgical methods on MMSE, MoCA and HAMD scores with right BG

To evaluate the effects of different surgical methods on patients' mental state, MMSE, MoCA, and HAMD scores were compared among patients with right BGH (Fig. 1). Significant differences were found in the different time points in each group (Z = 82.00, P = 0.001) (Table 7), with a significant difference in HAMD scores between 2 weeks and 1 month in group I. At 3 months after the operation, there were significant differences in MMSE scores among the three groups (Z = 6.886, P = 0.032), with a difference between groups I and III at α = 0.10 (Z = 2.228, P = 0.078). Significant differences in MoCA scores were found between the three groups at 2 weeks (Z = 8.772, P = 0.012) and 1 month (Z = 6.50, P = 0.039), with differences between groups I and III at 2 weeks and 1 month (P < 0.05). Furthermore, significant differences were observed between different time points in each group (Z = 77.975, P = 0.001), with significant differences between 3 months and 2 weeks and between 2 weeks and 1 month in group I.

**Table 7:** Effect of surgical methods on MMSE, MoCA and HAMD scores in the right BG[*M* (IQR), n = 41].

	I (22)	II (10)	III (9)	SUM	*Z*	*P*
MMSE (n = 41)						
2 week	[1.5(9.0)]^▲^	[9.0(12.0)]	[11.0(15.0)]		5.497	0.064
1 month	[9.5(17.0)]^◎^	[16.0(16.0)]	[14.0(14.0)]		4.669	0.097
3 month	[12.0(21.0)]^#^	[21(12.0)]^#^	[19.0 (13.0)]^#^*		6.886	0.032
*Z*	41.659	20.00	18.00	79.342		
*P*	0.001	0.001	0.001	0.001		
MOCA (n = 41)						
2 week	[0(3.0)]^▲^	[3.0((4.0)]	[5.0(7.0)]*		8.772	0.012
1 month	[5.5(6.0)]^◎^	[8.0(8.0)]	[9.0 (15.0)]*		6.500	0.039
3 month	[12.0(16.0)]^#^	[19.0 (11.0)]^#^	[16.0(14.0)]^#^		5.509	0.064
*Z*	41.952	20.00	16.222	77.975		
*P*	0.001	0.001	0.001	0.001		
HAMD (n = 41)						
2 week	[23.5(10.0)]^▲^	[24.0(6.0)]	[27.0(7.0)]		0.879	0.644
1 month	[16.0(4.0)]^◎^	[15.0 (4.0)]	[13.0(5.0)]		3.179	0.204
3 month	[9.0(4.0)]^#^	[8.5((3.0)]^#^	[7.0 (4.0)]^#^		0.078	0.702
*Z*	44.00	20.00	18.00	82.00^@^		
*P*	0.001	0.001	0.001	0.001^@^		

MMSE scores were significantly different at the α = 0.10 level compared to the I group, and MoCA and HAMD scores were significantly different at the *α* = 0.05 level compared to the I group. Friedman test analysis showed: ^▲^showed significant difference at α = 0.05 level between 2-week time point and 1-month time point; ^◎^showed significant difference at *α* = 0.05 level between 1-month time point and 3-month time point; ^#^showed significant difference at *α* = 0.05 level between 3-month time point and 2-week time point.

### HAMD, MMSE and MoCA scores at different postoperative times in different haemorrhage locations

To evaluate the effect of bleeding location (right BGHs, right BGBVHs, CLHs, VIHs, and cerebellar hemorrhages) on HAMD, MMSE, and MoCA scores at different time points, patients with varying degrees of cognitive impairment at 2 weeks and 1 and 3 months post-surgery were included. Mauthly’s spherical symmetry test analysis revealed a correlation between repeated measurement data, and multiple variance analysis showed a significant difference in HAMD scores among the five groups (F = 3.125, P = 0.019), with pairwise comparisons showing patients with right BGHs had the highest HAMD score at 2 weeks. MMSE scores showed no significant differences among the five groups, but there were significant differences at different time points in each group (Z = 177.287, P = 0.001), with pairwise comparisons showing gradual improvement in mental state at 1 and 3 months after onset. MoCA scores showed significant differences among the five groups at 2 weeks and 1 month but not at 3 months, with pairwise comparisons showing significant differences in the right BGH and right BGBH groups compared to the cerebellar hemorrhage group at 2 weeks and 1 month. The cognitive function of patients in all five groups improved gradually over time with statistical significance (P < 0.001) (Table 8).

**Table 8:** HAMD, MMSE, MoCA scores at different postoperative times in different hemorrhage locations.

Group	n	2nd week	1st month	3rd month	Sum	*F/Z*	*P*
HAMD							
Right BGH	41	24.85 ± 5.530^▲^	15.34 ± 3.991^◎^	8.44 ± 3.099^#^		236.705	< 0.001
Right BGBVH	20	20.80 ± 5.095^▲^*	13.95 ± 3.720^◎^	7.30 ± 2.867^#^		85.57	< 0.001
CLH	18	19.20 ± 4.550^▲^*	13.40 ± 2.408^◎^	7.80 ± 3.271^#^		19.38	< 0.007
VIH	10	19.83 ± 6.688*	13.17 ± 4.829^◎^	6.44 ± 2.455^#^*		78.552	< 0.001
Cerebellum	5	22.70 ± 5.417^▲^	16.30 ± 5.438^◎^	8.50 ± 3.894^#^		164.913	< 0.001
Sum						270.409	< 0.001
*F*		3.686	1.459	1.616	3.125	(*F* = 1.860, *P* = 0.086)^★^	
*P*		0.008	0.221	0.177	0.019^@^		
MMSE [*M* (IQR)]							
Right BGH	41	[4.0 (11.5)]^▲^	[11.0 (16.5)]^◎^	[18.0 (15.5)]^#^		79.342	0.001
Right BGBVH	20	[1.5 (14.0)]^▲^	[8.0 (16.0)]^◎^	[15.0 (19.3)]^#^		37.081	0.001
CLH	18	[15.0 (19.5)]	[17.0 (26.5)]	[26.0 (26.5)]^#^		8.588	0.014
VIH	10	[7.0 (18.8)]^▲^	[13.0 (18.8)]^◎^	[25.5 (17.0)]^#^		33.091	0.001
Cerebellum	5	[16.0 (19.8)]	[20.0 (18.3)]	[28.0 (2.25)]^#^		19.158	0.001
Sum						176.606	0.001
*Z*		6.741	5.275	6.302			
*P*		0.150	0.260	0.178			
MoCA [*M* (IQR)]							
Right BGH	41	[3.0(3.0)]^▲▽※^	[7.0 (7.0)]^◎※^	[15.0 (11.0)]^#^		77.975	0.001
Right BGBVH	20	[0 (8.0)]^▲※^	[3.0 (10.0)]^◎▽※^	[16.0 (16.0)]^#^		37.52	0.001
CLH	18	[10.0 (19.0)]	[21 (23.0)]	[27.0 (27.0)]^#^		9.333	0.009
VIH	10	[5.5 (19.0)]^▲^	[10.5 (22.0)]^◎^	[21.5 (20.0)]^#^		33.114	0.001
Cerebellum	5	[10.5 (17.0)]^▲^	[19.5 (15.0)]^◎^	[26.0 (9.0)]^#^		20.00	0.001
Sum						177.287	0.001
*Z*		11.189	10.264	9.016			
*P*		0.025	0.036	0.061			

1. HAMD, @ indicates F- and P values for main effects, ^★^indicates F- and P values for interaction effects. ANOVA on repeated-measures data: ^▲^indicates that the 2nd week is significantly different from the 1st month at the α = 0.05 level. ^◎^indicates that the 1st month point is significantly different from the 3rd month point at the α = 0.05 level; ^#^indicates that the 3rd month and the 2nd week point are significantly different at the α = 0.05 level. LSD pairwise comparison: *indicates a significant difference at the α = 0.05 level with the right BGH group. MMSE, analysed by the Friedman test: ^▲^indicates a significant difference at *α* = 0.05 between 2 weeks and 1 month; indicates a significant difference at *α* = 0.05 between 1 and 3 months; ^#^indicates a significant difference at *α* = 0.05 between 3 months and 2 weeks. MoCA, as analysed by the Friedman test: ^▲^indicates a significant difference at *α* = 0.05 between the 2nd week and 1st month; indicates a significant difference at *α* = 0.05 between the 1st month and 3rd month; # indicates a significant difference at *α* = 0.05 between the 3rd month and 2nd week. Indicates a significant difference at the *α* = 0.05 level compared with the cerebellar hemorrhage group. ^※^Significant difference at the *α* = 0.10 level compared with the cerebellar hemorrhage group.

## Discussion

We found that patients with hypertensive cerebral hemorrhage had varying degrees of depression and cognitive impairment after surgery. The location of bleeding, limb dysfunction, and inability to return to society were found to be factors affecting depression. Cognitive impairment was related to bleeding location, GCS score, ADL score, and MoCA score. The less invasive hematoma puncture and external drainage procedure had less impact on cognitive function than craniotomy and decompressive craniectomy, but it is not suitable for massive cerebral hemorrhage. Early treatment for post-operative depression is important and antidepressants may improve patient prognosis, but the optimal time and duration of treatment are still unknown^21^.

There are several challenges in assessing depression and cognitive dysfunction in patients who have had hypertensive intracerebral hemorrhage, such as unconsciousness, non-cooperation, coma, and aphasia, which may make it difficult to obtain valid scores. In this study, only patients with normal language function were evaluated using MMSE, MoCA, and HAMD scales to assess depression and cognitive function. Patients were classified into normal and depressed groups based on their HAMD scores at 3 months, and various factors were compared between the two groups, including sex, age, visiting time, GCS score, brain herniation, bleeding location, and surgical method. Postoperative factors, such as blood pressure, rehabilitation status, complications, and ADL and FMA scores, were also assessed. The study found that there was a linear relationship between HAMD score and ADL (BI) and FMA scores in the depression group, mainly due to limb dysfunction affecting patients' mood, which is consistent with a previous study by Hai et al.^22^. FMA and ADL scores below ground mean that impaired physical mobility affects quality of life^23^. Result in reduced functional independence, social isolation, reduced social contacts can be a cause as well as a result of depression^24^. Predictors of depression in this study included preoperative NIHSS (high) and GCS (low) scores, as well as postoperative BI (low), mRS (high), and SSQOL (low) scores. Additionally, the ADL (BI) and NIHSS scores on the 15th day in the FAST test were found to be correlated with PSD on the 90th day^6^.

While there is no correlation between lesion site and PSD^25^, we found that the HAMD score in the right BGH group was higher than that in the right BGBVH, VIH, and CLH groups at 2 weeks and/or 3 months, suggesting that the lesion location is closely related to the severity of depression in the first 3 months after stroke. Physical disability and stroke severity are also related to PSD^26^. Approximately one quarter of cerebral hemorrhage patients develop post-intracerebral hemorrhage depression (PIHD)^6^. Additionally, two-thirds of patients with hypertensive cerebral hemorrhage in the basal ganglia develop depression following surgical clearance^22^, which is consistent with our findings. We found that the incidence of depression was more than half after 3 months the right basal ganglia hemorrhage operation. The trend of PSD is diverse, with some recovering and others becoming chronic^7^. According to the HDRS (HAMD) score, the most common specific symptom areas are depressed mood, worrying, general physical symptoms, sexual symptoms, and stopped working because of ICH^3^. Therefore, it is crucial to preserve and/or improve patients' limb motor function and reduce the occurrence of depression.

The prevalence of cognitive impairment post cerebral hemorrhage is 14–88%^27^. From our current study we found that there was a linear relationship among MMSE and GCS and ADL scores in the cognitive impairment group 3 months post-surgery. In addition, there was a linear relationship between MoCA score and bleeding location and ADL score in the cognitive impairment group. Our data suggests that the pre-operative GCS score is good indicator for the cognitive function post-surgery.

The influence of different cerebral hemorrhage sites on MMSE and MoCA scores was evaluated by comparing the scores of different groups at different time points. Significant differences were observed in MoCA scores at 2 weeks and 1 month among the 5 groups, with significant differences between the right basal ganglia hemorrhage (BGH) group and cerebellar hemorrhage group at 2 weeks, indicating that BGH contributes more to cognitive impairment. The presence of cognitive impairment following a stroke is associated with increased disability, dependence on activities of daily living, and higher rates of institutionalization^24^. The location and severity of the stroke play a critical role in determining the specific cognitive deficits observed in individuals^28^. Strokes affecting the frontal lobes are often associated with executive dysfunction, while strokes involving the temporal lobes can lead to memory impairment^29^. Our findings are consistent with previous studies suggesting that right basal ganglia damage impairs cognitive function by disrupting brain circuits and specific cognitive abilities^13,17,18^. Nerve projection fibers in the BG region through the anterior limb of the internal capsule are the fronto-pontine tract and anterior thalamic radiation, and damage to the BG interrupts the subcortical circulation of both the frontal and parietal lobes, leading to cognitive changes. Based on the above anatomical theory, there is a correlation between bleeding location and ADL score. However, there are currently no clinical intervention measures available for bleeding location and ADL score. Our goal was to promptly prevent/minimize bleeding area/size through surgical intervention to reduce potential post-surgical complications. The probability of cerebral hemorrhage growth drops steeply when the volume of cerebral hemorrhage is around 75 ml, which is generally within 0.5–3 h after the symptoms of cerebral hemorrhage appear^30^. This may explain why there was no significant difference in the "visiting time" between the depression and non-depression groups or between the cognitive impairment and non-cognitive impairment groups, as the median treatment time in this study was longer than 5 h.

In the current study, 73% CIH patients had cognitive dysfunction on the MMSE or MoCA scale. Among them, 16% patients with normal MMSE scores were classified as having cognitive impairment based on the MoCA scale. Our findings are in line with others, showing that 27% of patients with "normal" MMSE scores had an MoCA score of 25 or lower, indicating cognitive impairment^31^. However, the MMSE scoring system has some limitations, such as its insensitivity to language parameters and false positives, and it cannot differentiate between people with mild dementia and no dementia^14^.

The reliability of diagnosis was based on multiple critical value analysis, in which the AUC of the MMSE or MoCA was 0.736 (95% CI 0.718–0.767) or 0.846 (95% CI 0.823–0.868), respectively, suggesting that MoCA is superior to MMSE in detecting mild cognitive impairment MCI^32^, and MoCA is more valuable in predicting vascular cognitive dysfunction by Chinese clinicians (0.667 vs 0.626)^13^. Furthermore, MoCA has better sensitivity in screening cognitive impairment in elderly who live in the community, partially due to the lack of a ceiling effect in the MMSE^33^. MoCA scale is more sensitive than the MMSE in cognitive impairment score^34^.

The aim of surgery for these hemorrhagic patients is to "give the brain more space" rather than clear the bleeding, regardless of surgical approaches. Consequently, reduce intracranial pressure and "toxic" burden of intracerebral hemorrhage. Using HAMD, MoCA and MMSE scores systems, it was compared that the outcomes of different surgical approaches from these right BG patients on depression and cognitive function. There was no significant difference in HAMD score among these patients at any time point.

There was a significant difference in MMSE and scores between groups I and III at 3 months post-operation. Our findings suggest that minimally invasive procedures, and resulting in better cognitive function scores. This, in turn, helps to preserve brain function, conserve cognitive ability, and reduce depression. However, bone flap decompression is more appropriate for patients with massive cerebral hemorrhage and obvious midline deviation, as hematoma puncture and external drainage may not be suitable for such patients^35^.

This study found that patients with hypertensive cerebral hemorrhage had varying degrees of depression and cognitive impairment after surgery. Early treatment for post-operative depression is important and antidepressants may improve patient prognosis^36^, but the optimal time and duration of treatment are still unknown^35^.

Limitations of this study include a single-center design and a high rate of loss to follow-up, and strict exclusion criteria may limit the generalizability of our results to cerebral hemorrhage survivors with severe disability or aphasia. which resulted in a relatively small sample size. Patients with cerebral lobe hemorrhage and those who underwent lateral ventricle drainage were categorized into the skull drilling drainage group, which may have resulted in positional deviation when evaluating the surgical approach. Puncture hematoma aspiration and external drainage may not be suitable for patients with massive cerebral hemorrhage.

## Conclusion

In summary, early detection of depression and cognitive dysfunction, early use of drug intervention, and psychological rehabilitation, prior to the progression of consciousness disorder (i.e., before the GCS score decreases), along with appropriate surgical methods and post-operative care, may improve the treatment outcomes and prognosis of hypertensive cerebral hemorrhage patients.

## Data Availability

The datasets used and/or analysed during the current study available from the corresponding author on reasonable request.

## References

[1] Sun, X.-G. *et al.* Incidence and trends of stroke and its subtypes in Changsha, China from 2005 to 2011. *J. Clin. Neurosci.* **21**, 436–440. 10.1016/j.jocn.2013.04.028 (2014). 24169270 10.1016/j.jocn.2013.04.028

[2] Keep, R. F., Hua, Y. & Xi, G. Intracerebral haemorrhage: Mechanisms of injury and therapeutic targets. *Lancet Neurol.* **11**, 720–731. 10.1016/s1474-4422(12)70104-7 (2012). 22698888 10.1016/S1474-4422(12)70104-7 PMC3884550

[3] Christensen, M. C., Mayer, S. A., Ferran, J. M. & Kissela, B. Depressed mood after intracerebral hemorrhage: The FAST trial. *Cerebrovasc. Dis. (Basel, Switzerland)* **27**, 353–360. 10.1159/000202012 (2009). 10.1159/000202012 19218801

[4] Pasi, M., Sugita, L., Xiong, L. & Charidimou, A. Association of cerebral small vessel disease and cognitive decline after intracerebral hemorrhage. *Am. Acad. Neurol.* **96**, e182–e192. 10.1212/wnl.0000000000011050 (2021). 10.1212/WNL.0000000000011050 PMC7905779 33067403

[5] Li, X. *et al.* The significant effects of cerebral microbleeds on cognitive dysfunction: An updated meta-analysis. *PLoS One* **12**, e0185145. 10.1371/journal.pone.0185145 (2017). 28934304 10.1371/journal.pone.0185145 PMC5608335

[6] Avadhani, R. *et al.* Post-stroke depression in patients with large spontaneous intracerebral hemorrhage. *J. Stroke Cerebrovasc. Dis.* **30**, 106082. 10.1016/j.jstrokecerebrovas-dis.2021.106082 (2021). 34517296 10.1016/j.jstrokecerebrovasdis.2021.106082 PMC8532502

[7] Koivunen, R. J., Harno, H., Tatlisumak, T. & Putaala, J. Depression, anxiety, and cognitive functioning after intracerebral hemorrhage. *Acta Neurol. Scand.* **132**, 179–184. 10.1111/ane.12367 (2015). 25639837 10.1111/ane.12367

[8] Ayerbe, L., Ayis, S., Rudd, A. G., Heuschmann, P. U. & Wolfe, C. D. Natural history, predictors, and associations of depression 5 years after stroke: The South London Stroke Register. *Stroke* **42**, 1907–1911. 10.1161/strokeaha.110.605808 (2011). 21566241 10.1161/STROKEAHA.110.605808

[9] Creutzfeldt, C. J., Holloway, R. G. & Walker, M. Symptomatic and palliative care for stroke survivors. *J. Gen. Intern. Med.* **27**, 853–860. 10.1007/s11606-011-1966-4 (2012). 22258916 10.1007/s11606-011-1966-4 PMC3378740

[10] Sun, G. *et al.* The rule of brain hematoma pressure gradient and its influence on hypertensive cerebral hemorrhage operation. *Sci. Rep.* **11**, 4599. 10.1038/s41598-021-84108-w (2021). 33633221 10.1038/s41598-021-84108-w PMC7907243

[11] Zhao, Z. *et al.* Assessment of the effect of short-term factors on surgical treatments for hypertensive intracerebral haemorrhage. *Clin. Neurol. Neurosurg.* **150**, 67–71. 10.1016/j.clineuro.2016.08.023 (2016). 27596750 10.1016/j.clineuro.2016.08.023

[12] Guo, W. *et al.* Comparison of endoscopic evacuation, stereotactic aspiration, and craniotomy for treatment of basal ganglia hemorrhage. *J. Neurointerv. Surg.* **12**, 55–61. 10.1136/neurintsurg-2019-014962 (2020). 31300535 10.1136/neurintsurg-2019-014962 PMC6996102

[13] Zeng, K. *et al.* Relationship between EEG beta power abnormality and early diagnosis of cognitive impairment post cerebral hemorrhage. *Clin. EEG Neurosci.* **44**, 203–208. 10.1177/1550059412471336 (2013). 23676378 10.1177/1550059412471336

[14] Monroe, T. & Carter, M. Using the Folstein Mini Mental State Exam (MMSE) to explore methodological issues in cognitive aging research. *Eur. J. Ageing* **9**, 265–274. 10.1007/s10433-012-0234-8 (2012). 28804426 10.1007/s10433-012-0234-8 PMC5547414

[15] Yakushiji, Y. *et al.* Basal ganglia cerebral microbleeds and global cognitive function: The Kashima Scan Study. *J. Stroke Cerebrovasc. Dis.* **24**, 431–439. 10.1016/j.jstrokecerebrovasdis.2014.09.015 (2015). 25516488 10.1016/j.jstrokecerebrovasdis.2014.09.015

[16] Guerrero-Berroa, E. *et al.* The MMSE orientation for time domain is a strong predictor of subsequent cognitive decline in the elderly. *Int. J. Geriatr. Psychiatry* **24**, 1429–1437. 10.1002/gps.2282 (2009). 19382130 10.1002/gps.2282 PMC2919210

[17] Zabyhian, S. *et al.* Cognitive function, depression, and quality of life in patients with ruptured cerebral aneurysms. *Iran. J. Neurol.* **17**, 117–122 (2018). 30886678 PMC6420689

[18] Tveiten, A., Ljøstad, U., Mygland, Å. & Naess, H. Functioning of long-term survivors of first-ever intracerebral hemorrhage. *Acta Neurol. Scand.* **129**, 269–275. 10.1111/ane.12185 (2014). 24444381 10.1111/ane.12185

[19] Bagby, R. M., Ryder, A. G., Schuller, D. R. & Marshall, M. B. The Hamilton Depression Rating Scale: Has the gold standard become a lead weight?. *Am. J. Psychiatry* **161**, 2163–2177. 10.1176/appi.ajp.161.12.2163 (2004). 15569884 10.1176/appi.ajp.161.12.2163

[20] Sharp, R. The Hamilton rating scale for depression. *Occup. Med. (Oxford, England)* **65**, 340. 10.1093/occmed/kqv043 (2015). 10.1093/occmed/kqv043 25972613

[21] Salter, K. L., Foley, N. C., Zhu, L., Jutai, J. W. & Teasell, R. W. Prevention of poststroke depression: Does prophylactic pharmacotherapy work?. *J. Stroke Cerebrovasc. Dis.* **22**, 1243–1251. 10.1016/j.jstrokecerebrovasdis.2012.03.013 (2013). 22554569 10.1016/j.jstrokecerebrovasdis.2012.03.013

[22] Hai, J., Zhang, L., Wang, F., Wan, J. F. & Pan, Q. G. Quality of life with special respect to depression after surgical treatment of hypertensive basal ganglia hemorrhage. *Neurol. India* **58**, 74–77. 10.4103/0028-3886.60403 (2010). 20228468 10.4103/0028-3886.60403

[23] Erban, P. *et al.* Long-term outcome after hemicraniectomy for space occupying right hemispheric MCA infarction. *Clin. Neurol. Neurosurg.* **108**, 384–387. 10.1016/j.clineuro.2005.06.008 (2006). 16137824 10.1016/j.clineuro.2005.06.008

[24] Elendu, C. *et al.* Stroke and cognitive impairment: Understanding the connection and managing symptoms. *Ann. Med. Surg. (Lond.)* **85**, 6057–6066. 10.1097/ms9.0000000000001441 (2023). 38098605 10.1097/MS9.0000000000001441 PMC10718363

[25] Carson, A. J. *et al.* Depression after stroke and lesion location: A systematic review. *Lancet* **356**, 122–126. 10.1016/s0140-6736(00)02448-x (2000). 10963248 10.1016/S0140-6736(00)02448-X

[26] Rajashekaran, P., Pai, K., Thunga, R. & Unnikrishnan, B. Post-stroke depression and lesion location: A hospital based cross-sectional study. *Indian J. Psychiatry* **55**, 343–348. 10.4103/0019-5545.120546 (2013). 24459304 10.4103/0019-5545.120546 PMC3890916

[27] Donnellan, C. & Werring, D. Cognitive impairment before and after intracerebral haemorrhage: A systematic review. *Neurol. Sci.* **41**, 509–527. 10.1007/s10072-019-04150-5 (2020). 31802344 10.1007/s10072-019-04150-5

[28] Nys, G. M. *et al.* Cognitive disorders in acute stroke: Prevalence and clinical determinants. *Cerebrovasc. Dis. (Basel, Switzerland)* **23**, 408–416. 10.1159/000101464 (2007). 10.1159/000101464 17406110

[29] Hachinski, V. *et al.* National institute of neurological disorders and stroke-Canadian Stroke Network vascular cognitive impairment harmonization standards. *Stroke* **37**, 2220–2241. 10.1161/01.Str.0000237236.88823.47 (2006). 16917086 10.1161/01.STR.0000237236.88823.47

[30] Al-Shahi Salman, R. *et al.* Absolute risk and predictors of the growth of acute spontaneous intracerebral haemorrhage: A systematic review and meta-analysis of individual patient data. *Lancet Neurol.* **17**, 885–894. 10.1016/s1474-4422(18)30253-9 (2018). 30120039 10.1016/S1474-4422(18)30253-9 PMC6143589

[31] Aggarwal, A. & Kean, E. Comparison of the Folstein Mini Mental State Examination (MMSE) to the Montreal Cognitive Assessment (MoCA) as a cognitive screening tool in an inpatient rehabilitation setting. *Neurosci. Med.*10.4236/nm.2010.12006 (2010).

[32] Ciesielska, N. *et al.* Is the Montreal Cognitive Assessment (MoCA) test better suited than the Mini-Mental State Examination (MMSE) in mild cognitive impairment (MCI) detection among people aged over 60? Meta-analysis. *Psychiatr. Polska* **50**, 1039–1052. 10.12740/pp/45368 (2016). 10.12740/PP/45368 27992895

[33] Jia, X. *et al.* A comparison of the Mini-Mental State Examination (MMSE) with the Montreal Cognitive Assessment (MoCA) for mild cognitive impairment screening in Chinese middle-aged and older population: A cross-sectional study. *BMC Psychiatry* **21**, 485. 10.1186/s12888-021-03495-6 (2021). 34607584 10.1186/s12888-021-03495-6 PMC8489046

[34] Koski, L. Validity and applications of the Montreal cognitive assessment for the assessment of vascular cognitive impairment. *Cerebrovasc. Dis. (Basel, Switzerland)* **36**, 6–18. 10.1159/000352051 (2013). 10.1159/000352051 23920318

[35] Yao, Z., Ma, L., You, C. & He, M. Decompressive craniectomy for spontaneous intracerebral hemorrhage: A systematic review and meta-analysis. *World Neurosurg.* **110**, 121–128. 10.1016/j.wneu.2017.10.167 (2018). 29129764 10.1016/j.wneu.2017.10.167

[36] Liang, J. *et al.* Effects of noninvasive brain stimulation combined with antidepressants in patients with poststroke depression: A systematic review and meta-analysis. *Front. Pharmacol.* **13**, 887115. 10.3389/fphar.2022.887115 (2022). 35662704 10.3389/fphar.2022.887115 PMC9160966

